# Fertility preservation in female classic galactosemia patients

**DOI:** 10.1186/1750-1172-8-107

**Published:** 2013-07-16

**Authors:** Britt van Erven, Cynthia S Gubbels, Ron J van Golde, Gerard A Dunselman, Josien G Derhaag, Guido de Wert, Joep P Geraedts, Annet M Bosch, Eileen P Treacy, Corrine K Welt, Gerard T Berry, M Estela Rubio-Gozalbo

**Affiliations:** 1 Department of Pediatrics and Department of Genetic Metabolic Diseases Laboratory, Maastricht University Medical Center, Maastricht, the Netherlands; 2 GROW, Research school for Oncology and Developmental Biology, Maastricht University, Maastricht, the Netherlands; 3 Department of Clinical Genetics, Maastricht University Medical Center, Maastricht, the Netherlands; 4 Currently: The Manton Center for Orphan Disease Research, Division of Genetics, Boston Children’s Hospital, Harvard Medical School, Boston, USA; 5 Department of Obstetrics and Gynecology, Maastricht University Medical Center, Maastricht, the Netherlands; 6 Department of Health, Ethics & Society, Maastricht University, Maastricht, the Netherlands; 7 Department of Pediatrics, Academic Medical Center, Amsterdam, the Netherlands; 8 National Centre for Inherited Metabolic Disorders, Children’s University Hospital, Trinity College, Dublin, Ireland; 9 Reproductive Endocrine Unit, Massachusetts General Hospital, Harvard Medical School, Boston, USA; 10 The Manton Center for Orphan Disease Research, Division of Genetics, Boston Children’s Hospital, Harvard Medical School, Boston, USA

**Keywords:** Classic galactosemia, GALT deficiency, Primary ovarian insufficiency, POI, Fertility preservation, Cryopreservation

## Abstract

Almost every female classic galactosemia patient develops primary ovarian insufficiency (POI) as a diet-independent complication of the disease. This is a major concern for patients and their parents, and physicians are often asked about possible options to preserve fertility. Unfortunately, there are no recommendations on fertility preservation in this group. The unique pathophysiology of classic galactosemia with a severely reduced follicle pool at an early age requires an adjusted approach. In this article recommendations for physicians based on current knowledge concerning galactosemia and fertility preservation are made. Fertility preservation is only likely to be successful in very young prepubertal patients. In this group, cryopreservation of ovarian tissue is currently the only available technique. However, this technique is not ready for clinical application, it is considered experimental and reduces the ovarian reserve. Fertility preservation at an early age also raises ethical questions that should be taken into account. In addition, spontaneous conception despite POI is well described in classic galactosemia. The uncertainty surrounding fertility preservation and the significant chance of spontaneous pregnancy warrant counseling towards conservative application of these techniques. We propose that fertility preservation should only be offered with appropriate institutional research ethics approval to classic galactosemia girls at a young prepubertal age.

## Introduction

Almost every female patient with classic galactosemia, an inborn error of galactose metabolism, has primary ovarian insufficiency (POI) and is confronted with the struggle that reduced chances of having children presents [1]. Patients and/or their parents often approach physicians with questions regarding fertility preservation. Answering these questions, however, remains difficult, due to the unique and yet to be determined underlying pathophysiological mechanisms of POI as well as the lack of experience with fertility preservation techniques in general and in girls with classic galactosemia in particular.

Classic galactosemia (ORPHA79239) is caused by deficient activity of galactose-1-phosphate uridyl transferase (GALT), as a result of mutations in the *GALT* gene located on chromosome 9p13. GALT is the second of the three enzymes in the Leloir pathway, the main pathway of galactose metabolism. The incidence of classic galactosemia varies between 1:16.000 [2] and 1:60.000 [3] in Western countries. Galactose is needed for energy metabolism and glycosylation of complex molecules. It may be derived from exogenous (dietary) sources, most importantly lactose from dairy products, or endogenous production. Deficiency of the GALT enzyme leads to accumulation of galactose and its metabolites and results in secondary glycosylation abnormalities. Patients usually present in the first weeks of life with signs of liver and renal disease, cataracts and an Escherichia coli sepsis. Diagnostic tests include elevated galactose and galactitol in body fluids, elevated Gal-1-P in erythrocytes, severely diminished enzyme activity in erythrocytes and mutations in the *GALT* gene. A galactose-restricted diet quickly resolves the early signs, but cannot prevent the development of later-onset complications, such as cognitive impairment, neurological sequelae, bone health abnormalities, and, in female patients, POI with subsequent infertility.

Although POI in classic galactosemia represents a major concern for these patients and/or their parents [4], there are no published recommendations concerning fertility preservation in this group. The current guidelines in patients with cancer [5-10] may be useful in assessing the risk-benefit ratio for girls with galactosemia. However, unlike cancer patients, the mechanisms causing ovarian dysfunction are unknown and the timing of onset may begin as early as prenatally [1,11-14]. Therefore, intervening when the patient reaches adulthood may be too late. As a consequence, applying fertility preservation options in very young girls raises medical, legal and ethical questions. Furthermore, spontaneous pregnancies do occur in this disease, demonstrating that conception is possible in some patients.

The aim of this paper is to discuss the different aspects of fertility loss in female classic galactosemia patients from a multidisciplinary perspective, and to propose recommendations for fertility preservation, taking into account pathophysiology, chances of spontaneous pregnancies and ethical issues. These recommendations are based on our experiences with classic galactosemia patients and the currently available literature.

### Pathophysiology

Symptoms of POI differ between affected women, varying from subfertility, to early development of irregular menstrual cycles and infertility, to primary amenorrhea and absence of spontaneous puberty [15]. The cause of POI in classic galactosemia is not yet understood. Several mechanisms have been postulated, including direct toxicity of metabolites (i.e. galactose-1-phosphate), altered gene expression, or aberrant function of hormones and or receptors due to glycosylation abnormalities [1,16-18]. It is also possible that not one, but several mechanisms act in unison to cause POI in classic galactosemia.

In general, POI can be caused by either the formation of a smaller primordial follicle pool or more rapid loss of primordial follicles [15] and there is evidence for both mechanisms in classic galactosemia. In classic galactosemia there is some evidence that the follicle pool at birth is as large as in girls without this disease. In two galactosemic neonates, morphologically normal ovaries with abundant oocytes and normal folliculogenesis have been reported [19,20]. In another patient, ultrasound showed apparently normal ovaries at age 7 [21]. These studies suggest that the primordial follicle complement is normal initially, but comprehensive evidence is lacking.

Histological findings show that the ovaries contain a strongly reduced number of follicles at older, but variable ages. Histological findings in adolescents (aged 16 or 17 years) reveal a strongly reduced number of follicles, varying from far fewer follicles than expected for age [14] to almost complete absence [22-24]. Data in younger adolescents or children are lacking. Histological findings in two adults showed absence of ovarian parenchyma [25] and an extremely reduced number of follicles in one subject [26], and a follicle number within the normal range in the other [26]. Ovarian size and volume in patients aged 9–21 years assessed with MRI were comparable to those found in postmenopausal controls [27]. Ultrasound or laparoscopy/laparotomy in patients during adolescence or adulthood often shows hypoplastic [24,28,29] or streak ovaries [22,23,25,30], consistent with the absence of follicles. Of note, streak ovaries were also found during adolescence in the girl with apparently normal ovaries at age 7 [21]. Taken together, it is possible that a normal complement of primordial follicles forms, but undergoes atresia more rapidly and that the ovaries can be severely damaged in girls at very young prepubertal age.

There is also evidence for defective follicle maturation, which may result from a paucity of follicles able to respond to stimulation or inability of the follicle to respond to gonadotropins in galactosemia. The few follicles present in the ovaries of classic galactosemia patients are mainly of the primordial type, and maturing follicles are rarely seen [14,22,23,26,28]. Consistent with the absence of estrogen production due to absent ovarian activity [26,27,31], hypoplastic prepubertal uteri were observed in patients who did not receive estrogen supplementation [21-23,29,30].

Animal studies suggest that galactose might already have a direct toxic effect in fetal life. After feeding pregnant and lactating Sprague–Dawley rats a high galactose diet, the offspring had a significant and striking reduction in the number of small follicles, [11], a reduced number of primordial germ cells (PGCs) and a consequently smaller gonadal size [32] suggesting impaired germ cell migration, and a deficient complement of follicles in some of the exposed animals [33].

An accelerated loss of follicles during life has also been suggested in several studies. Lai et al. found that the number of apoptotic granulosa cells of maturing follicles was higher in Sprague–Dawley rats fed a high galactose diet than in animals receiving normal chow [34]. The same has been concluded in a recent study by Banerjee et al. [35]. Their results in isolated granulosa cells suggest that galactose exposure leads to an increased expression of p53, a protein mediating intrinsic death pathways in cells [36], consequently causing follicular atresia. The authors also suggest that attenuated FSH bioactivity is the underlying cause of the increased p53 expression. The latter is not consistent with failure of follicle response to exogenous FSH [27,37].

Some animal studies also point to a compromised maturation. A high galactose diet significantly decreased the number of healthy growing and antral follicles, whereas the number of primordial or total atretic follicles was not affected [38], although other studies suggested that the primordial follicle count was lower [11]. Similarly, other studies using the same model demonstrated a decreased ovulatory response [33,39,40], evidenced by reduced numbers of corpora lutea.

Both case reports of neonates compared to older girls and the animal studies suggest that galactosemia results in increased follicle apoptosis with an accelerated follicle loss of either primordial follicles or maturing follicles as the cause of POI [34,35,38]. It might be very difficult to determine whether the large follicle pool present at birth, consisting of millions of primordial follicles, is reduced in classic galactosemia patients unless the decrease is marked. Animal models indicate a prenatal toxic effect of galactosemia but these animal studies, in which animals are fed a high galactose diet, might not represent human classic galactosemia completely. Although the histological and imaging data are very limited and call for a tedious conclusion, taken together, there seems to be a rapid decline in the number of primordial follicles either in fetal life or in early life in this disease, which has to be taken into account when discussing fertility preservation.

### Spontaneous pregnancies in women with classic galactosemia

Over the past years it has become increasingly clear that spontaneous pregnancies in classic galactosemia occur, and occur more frequently than previously thought. In a cohort of 22 patients with classic galactosemia and POI, 9 patients tried to conceive, of which 4 succeeded (44 percent) [41]. Although significant, the small sample size warrant further studies in a larger cohort. Many patients were told in the past that they had no chance of conceiving and therefore may not have tried, which makes it difficult to determine the exact chance of spontaneous pregnancy in classic galactosemia. It is important that patients are aware of the occurrence of spontaneous pregnancies [2,31,41-43], allowing them both to try to conceive spontaneously and to avoid unplanned pregnancies. The World Health Organization defines infertility as a failure to achieve spontaneous pregnancy within twelve months or more [44]. Therefore we advise physicians to encourage their patients to try to achieve spontaneous pregnancy for at least this period of one year. We also advise physicians to explain that pregnancy should not be postponed unnecessarily to increase chances of conception. Importantly, classic galactosemia women became pregnant despite postmenopausal FSH levels, extremely low estrogen concentrations, undetectable Müllerian Inhibitory Substance (MIS) or anti-Müllerian hormone (AMH) levels [31,45] and the severe Q188R/Q188R genotype [2,41,43]. Therefore, genotype, enzyme activity in erythrocytes, and measurement of markers of ovarian reserve do not appear accurate predictors of pregnancy chances in this patient population. Besides, the ovaries of prepubertal girls are very small, even in healthy girls, limiting the relevance of imaging techniques in these patients. Of note, most of the women that became pregnant spontaneously have gone through normal puberty and reached menarche spontaneously, indicating that these might be predictive factors for an increased chance of spontaneous conception [41].

### Fertility preservation in classic galactosemia patients

Importantly, fertility preservation data are mainly derived from cancer patients. The ovaries of a girl affected with non-ovarian cancer are healthy when there is a need for fertility preservation. In contrast, the ovaries of classic galactosemia girls are probably already damaged at an early age. Therefore, the success rate of the procedures is probably lower in galactosemic girls. Perhaps the most important issue to discuss before choosing to apply fertility preservation techniques is the fact that fertility preservation does not guarantee future pregnancy. A thorough evaluation including the abovementioned chance of spontaneous pregnancy and the risks and benefits needs to be taken into account. Three fertility preservation procedures currently offered to girls and women in need of fertility preservation are: a. ovarian tissue cryopreservation, b. mature oocyte cryopreservation and/or c. embryo cryopreservation. The latter two techniques require ovarian stimulation with FSH, whereas cryopreservation of ovarian tissue does not.

#### a. Ovarian tissue cryopreservation

In ovarian tissue cryopreservation, one ovary is harvested through laparoscopy and the cortex tissue is processed into cortical strips and frozen. When the patient is ready to conceive, the tissue is transplanted at either an orthotopic or a heterotopic location in the body. Currently, twenty healthy infants have been born worldwide as a result of autotransplantation of the cryopreserved tissue [46]. All mothers were women with no history of ovarian problems who were treated with chemotherapy after cancer diagnosis. However, the method is still experimental and consensus is lacking on the amount of tissue that needs to be harvested for cryopreservation [47,48]. The major risk of this technique is that the graft may not survive due to ischemic damage, which leads to massive follicular death [49,50]. As primordial follicles are more resistant to this damage [49], the chance of restoring fertility is related to the number and quality of the primordial follicles in the transplanted cortical tissue [51]. Therefore, it is possible that the quantity of ovarian tissue removed should be influenced by the expected probability of POI [47]. Another disadvantage of cryopreservation of ovarian tissue is that this technique might lower the chance of spontaneous pregnancy, as a result of reduction of the remaining ovarian pool. Moreover, it is also possible that the transplanted tissue is subject to the same detrimental factors that cause POI in this patient group.

#### b. Mature oocyte cryopreservation

In oocyte cryopreservation the patient’s ovaries are stimulated with exogenous FSH, the growing follicles are punctured transvaginally and the oocytes are extracted from the follicles. The metaphase II oocytes are frozen using vitrification, a technique in which high initial concentrations of cryoprotectant and ultra-rapid cooling are used to create a glass-like state in a cell without forming ice crystals. Once the patient desires to conceive, the oocytes are warmed and consequently fertilized in a petri dish using a partner’s or donor’s semen (intracytoplasmatic sperm injection, ICSI). The procedure of mature oocyte cryopreservation is no longer considered experimental by multiple international reproduction societies, including the American Society for Reproductive Medicine (ASRM) [52]. A recent review of the literature and meta-analysis suggested that fertilization and pregnancy rates were similar with vitrified/warmed oocytes and fresh oocytes [52]. However, a limitation of this analysis is that the majority of data is derived from studies with oocytes obtained from healthy, young donors. The current recommendations by the ASRM state that oocyte cryopreservation is recommended for patients facing infertility due to chemotherapy or other gonadotoxic therapies, but that more widespread clinic-specific data on the safety and efficacy of oocyte cryopreservation in donor populations are needed before universal donor oocyte banking can be recommended. Therefore, its use in patients with galactosemia is as yet unclear.

#### c. Embryo cryopreservation

A third fertility preservation option is embryo cryopreservation, a well-established and successful technique. This procedure consists of ovarian stimulation, transvaginally harvesting cumulus oocyte complexes and extraction of oocytes. The oocytes are fertilized *in vitro* using a partner’s or donor’s semen. The fertilized oocytes also develop *in vitro* to the embryo stage and are then cryopreserved by means of vitrification or slow freezing. Once the patient desires to conceive embryos are warmed and implanted in the uterus. The embryo survival rate is approximately 90% when vitrification is used [53]. Pregnancy rates in regular IVF programs are on average 30% [54].

Since all fertility preservation techniques depend on the ovarian reserve [55], and considering the apparent rapid follicle pool decline in classic galactosemia, fertility preservation in these patients may need to take place during infancy or early childhood, seriously limiting the options. Techniques requiring ovarian stimulation are not suitable for prepubertal girls because of absence of maturation of the hypothalamic-hypophyseal-ovarian axis [56], leaving cryopreservation of ovarian tissue the only available technique for this age group. Fertility preservation in older, postpubertal classic galactosemia patients is likely to be unsuccessful as the ovarian reserve may be poor. Embryo cryopreservation and oocyte cryopreservation are, in theory, the best options in these patients. However, both techniques require ovarian stimulation. Ovarian stimulation in fifteen classic galactosemia patients, aged 15 to 36 years demonstrated poor estradiol production in all but one [27]. Therefore, the severely reduced follicle pool limits the use of cryopreservation of embryos and mature oocytes [1]. It might be an option for those young classic galactosemic women with a sufficient ovarian reserve, but these women probably are the women whose chances of spontaneous conception are highest.

If fertility preservation is not a reasonable option, or if pregnancy does not occur despite fertility preservation, another option is adoption or the use of donor oocytes to achieve pregnancy. The donor can be anonymous or can be a direct donor, such as a mother or sister. Mothers of galactosemic girls frequently propose to donate oocytes for their daughters. It may be necessary to cryopreserve the mother’s oocytes for the future if the patient is not ready for children at the time of donation. The ethical issues resulting from intra familial donation are discussed below.

In the future, other fertility preservation techniques might become available. As an alternative for mature oocyte cryopreservation, cryopreservation of oocytes extracted from immature follicles has been evaluated (in vitro maturation, IVM). Another fertility preservation technique that is currently being evaluated and might be an option in the future is freezing and transplantation of the entire ovary including its vascular system [57,58]. Also the possible existence of oogonial stem cells [59] and induced pluripotent stem cells [60] might offer therapeutic options for the future.

### Ethical considerations in fertility preservation

Ethical problems arise in all young patients in need of fertility preservation, regardless of the underlying etiology. Girls are often too young to decide about fertility preservation themselves and therefore their parents play a crucial role in this process. One probably cannot predict with certainty how she herself or, in case of the parents, their daughter will think about a pregnancy in the future. The parent’s decision may not ultimately reflect the girl’s wishes when she becomes an adult [8]. In 2005, the American Society for Reproductive Medicine (ASRM) stated that fertility preservation can be applied in minors if this is in the best interest of the child and if the girl gives her assent [6]. If a girl is too young to give assent, parents may give consent if the procedure offers a net benefit for the child. Review of this decision by the ethics committee of a hospital or another independent body is in our opinion a must. The same criteria and advice hold true for fertility preservation techniques that are still considered experimental, though approved by the institutional ethics committee. Psychosocial guidance of the girl during the procedure and further discussion about fertility during adolescence and adulthood are very important. Moreover, legislation always plays a role in medical decision-making, but laws often differ between countries and may not be specific.

Cryopreservation of ovarian tissue, the most plausible fertility preservation option for this population, is still considered experimental and pregnancy rates resulting from this procedure are uncertain [9]. Recommending methods that are not yet established may offer false hope [1]. Therefore, the benefits and harms of every procedure should be discussed extensively, and chances of spontaneous pregnancy in this particular disease despite POI should be considered.

Intrafamilial oocyte donation might lead to ethical issues including psychosocial pressure on the donor and recipient, over-attachment of the donor to the offspring, and role confusion for the persons involved, including the resulting offspring [61]. The process of intrafamilial donation requires the help of multiple professionals, a thorough screening of the donor, psychological counseling of both the donor and the recipient and extensive informing about legal parenting relations [61,62].

## Discussion

As a consequence of the severe depletion of the follicle pool that probably occurs prenatally or early in childhood in classic galactosemia patients, fertility preservation in this group is only likely to be successful in very young patients. Oocyte cryopreservation after ovarian stimulation will likely not be successful in most women with classic galactosemia. It might be useful in women with a sufficient ovarian reserve who wish to postpone pregnancy. Ovarian tissue cryopreservation, the only available technique at a young age, has several important disadvantages, including the experimental character of the procedure and the reduced chances of spontaneous pregnancy. Therefore, the technique should probably not yet be offered in a clinical setting, but could be considered on research basis.

In general, classic galactosemia patients may have below-average cognitive functioning [2,63-65] and social-emotional problems [66,67], which might affect the ability to raise children. However, the severity of these impairments will not be clear at the time of fertility preservation, since this can change over time [68]. At the time of fertility preservation, physicians should explain to the patient’s parents that some patients might not develop enough parental competency and that this might prevent the use of cryopreserved tissue. In adulthood, when the cognitive and ovarian phenotype of the patient is clear and when psychological tests have been performed, the adult patient can decide if she wishes to have children and if she wants to use the cryopreserved tissue in order to achieve this. At that time, physicians and the ethics committee of the hospital or another independent body should evaluate whether the patient has adequate parental competency, in case this is doubtful. This is consistent with other fertility preservation procedures, for instance in pediatric cancer patients [10].

Physicians confronted with questions regarding POI and fertility preservation in classic galactosemia, should emphasize that pregnancy has been repeatedly reported despite POI and they should not discourage patients to try to conceive spontaneously. Noteworthy, girls with a spontaneous puberty are likely to have higher chances of spontaneous pregnancy. Furthermore the risk of postponing pregnancy needs to be discussed as well, since in galactosemic women, as in any woman, fertility declines with age, lowering the risk of spontaneous conception. However, due to the unknown course of ovarian function and the unknown pathophysiological mechanism of POI in classic galactosemia, decisions about fertility and fertility preservation remain difficult for patients, parents and physicians.

### Conclusions and recommendations

Based on the current knowledge about classic galactosemia and its pathophysiology, we recommend that fertility preservation should not be offered to these patients as common practice (see the ‘Our recommendations regarding fertility preservation in female classic galactosemia patients’ subsection). If it is part of institutional research ethics approved research it might be taken into careful consideration. Cryopreservation of ovarian tissue in young prepubertal girls is at this moment the procedure of choice. At a later age, estimated success rates are too low since the ovarian damage is probably already too prominent. Trying to achieve spontaneous pregnancy seems to be the best option for older girls and women.

Another option for classic galactosemic girls and women might be oocyte donation, both anonymous donation and intrafamilial donation. We currently do not recommend intrafamilial donation by the mother, as mentioned above. If in a special situation oocyte donation by the patient’s mother is considered, psychological screening and extensive counseling are required, according to previously mentioned guidelines [61].

Finally, we recommend engagement of an independent review board in the process of fertility preservation at minimum two points in the process: when the parental decision to preserve their daughter’s ovarian tissue is considered, but also when the patient wishes to use the preserved tissue, as the parental capacity of the patient should be reviewed.

#### Our recommendations regarding fertility preservation in female classic galactosemia patients

• Physicians should emphasize that spontaneous pregnancies occur in women with classic galactosemia, even after POI diagnosis.

• If fertility preservation is desired, cryopreservation at an early prepubertal age as a part of approved research currently seems to be the best option.

• The ethics committee of the hospital or another independent body should review the parent’s decision before the fertility preservation procedure.

• The ethics committee of the hospital or another independent body should be involved in the decision making surrounding the use of the cryopreserved material.

• If a patient desires pregnancy, a one-year window for attempting spontaneous pregnancy is advised to avoid unnecessary use of assisted reproductive techniques.

• Anonymous or intrafamilial oocyte donation might be another option for classic galactosemia patients if pregnancy does not occur.

## Competing interests

The authors declare that they have no competing interests.

## Authors’ contributions

BE, PhD on galactosemia, performed the study and drafted the manuscript. CG helped to draft the manuscript and gave critical comments. She has her PhD on fertility in galactosemia. RG and GD supervised the gynecological issues. JD gave critical comments, on the assisted reproductive techniques. GW coached the ethical aspect of the review. JG assisted us with critical comments as senior geneticist. AB, ET, GB, reviewed the manuscripts several times as galactosemia experts. CW critically assisted us in this study from her Reproductive Endocrine area of expertise. ER, PhD supervisor and lead of the galactosemia research group in Maastricht, designed, supervised and coordinated the study and the final version of this manuscript. All authors have read and approved the final manuscript.
